# Acetylation modification regulates GRP78 secretion in colon cancer cells

**DOI:** 10.1038/srep30406

**Published:** 2016-07-27

**Authors:** Zongwei Li, Ming Zhuang, Lichao Zhang, Xingnan Zheng, Peng Yang, Zhuoyu Li

**Affiliations:** 1 Institute of Biotechnology, Key Laboratory of Chemical Biology and Molecular Engineering of National Ministry of Education, Shanxi University, Taiyuan, 030006, China; 2 Department of Lymphoma and Myeloma, Division of Cancer Medicine, Center for Cancer Immunology Research, The University of Texas MD Anderson Cancer Center, Houston, Texas, USA; 3 General Surgical Department, Xinhua Hospital Affiliated to Shanghai Jiao Tong University School of Medicine, Shanghai, 200092, China; 4 Lineberger Comprehensive Cancer Center, University of North Carolina School of Medicine, Chapel Hill, North Carolina 27599, USA

## Abstract

High glucose-regulated protein 78 (GRP78) expression contributes to the acquisition of a wide range of phenotypic cancer hallmarks, and the pleiotropic oncogenic functions of GRP78 may result from its diverse subcellular distribution. Interestingly, GRP78 has been reported to be secreted from solid tumour cells, participating in cell-cell communication in the tumour microenvironment. However, the mechanism underlying this secretion remains elusive. Here, we report that GRP78 is secreted from colon cancer cells via exosomes. Histone deacetylase (HDAC) inhibitors blocked GRP78 release by inducing its aggregation in the ER. Mechanistically, HDAC inhibitor treatment suppressed HDAC6 activity and led to increased GRP78 acetylation; acetylated GRP78 then bound to VPS34, a class III phosphoinositide-3 kinase, consequently preventing the sorting of GRP78 into multivesicular bodies (MVBs). Of note, we found that mimicking GRP78 acetylation by substituting the lysine at residue 633, one of the deacetylated sites of HDAC6, with a glutamine resulted in decreased GRP78 secretion and impaired tumour cell growth *in vitro*. Our study thus reveals a hitherto-unknown mechanism of GRP78 secretion and may also provide implications for the therapeutic use of HDAC inhibitors.

Cells communicate and exchange signals through different secretory mechanisms, such as the default ER-Golgi secretion route, plasma membrane-derived microparticles (MPs) and exosomes^1^,^2^,^3^. Among these, extracellular vesicles, including MPs and exosomes, have attracted increasing attention as efficient vehicles for the transfer of membrane and cytosolic proteins, lipids, and RNA between cells^4^. MPs, a heterogeneous population of vesicles of sizes ranging from 100 nm to 1000 nm in diameter, bud directly from the plasma membrane and are characterized by phosphatidylserine (PS) exposure at the outer membrane leaflet^2^,^4^. Exosomes originate via inward budding of endosomal membranes, resulting in the formation of multivesicular bodies (MVBs) that fuse with the plasma membrane to release the exosomes into the extracellular space. Exosomes are of a homogenous size ranging from 40 nm to 100 nm and a density of 1.13 g/ml, and in contrast to MPs, these vesicles have low PS exposure but are rich in tetraspanin proteins, such as CD63 4,5,6. There is growing evidence that exosomes participate in recruitment and reprogramming of constituents associated with the tumour microenvironment^7^,^8^,^9^,^10^,^11^.

Glucose-regulated protein 78 (GRP78) is commonly induced in poorly perfused solid tumours by microenvironmental factors such as hypoxia, acidosis and glucose deficiency^12^. High levels of GRP78 contribute to the acquisition of phenotypic cancer hallmarks, including apoptosis resistance, immune escape, metastasis and angiogenesis^13^. However, acetylation of GRP78 induced by HDAC inhibitors in the ER is associated with activation of a lethal UPR in human breast cancer cells^14^. In addition to localizing in the ER, GRP78 is also present in the plasma membrane, cytoplasm, mitochondria and nucleus of tumour cells^15^. Specifically, GRP78 is secreted by tumour cells and blocks the antiangiogenic activity of bortezomib^16^. We recently demonstrated that secreted GRP78 prompts tumour cell proliferation and mesenchymal stem cell differentiation in an autocrine/paracrine manner^17^,^18^. However, the mechanism underlying GRP78 secretion by cancer cells remains unclear.

Found in the ER, plasma membrane and cytoplasm in tumour cells, GRP78 can theoretically be released through ER-Golgi, MP and exosome secretory routes. In the present study, we provide evidence that GRP78 is secreted from colon cancer cells via exosomes. HDAC inhibitors impair this process by inducing GRP78 aggregation in the ER, and further mechanistic study shows that HDAC inhibitors increase GRP78 K633 acetylation by blocking HDAC6 activity. Acetylated GRP78 dissociates from HDAC6 and binds to the VPS34 complex. Importantly, the K633Q mutation, which mimics K633 acetylation, leads to decreased GRP78 secretion and retards tumour cell growth *in vitro* and *in vivo*. Because HDAC6 is comparatively highly expressed in colon cancer cells, we propose that high HDAC6 expression in tumours gives rise to a lowly acetylated but highly secreted form of GRP78.

## Results

### GRP78 is secreted via membrane vesicles from colon cancer cells

**Figure 1:**
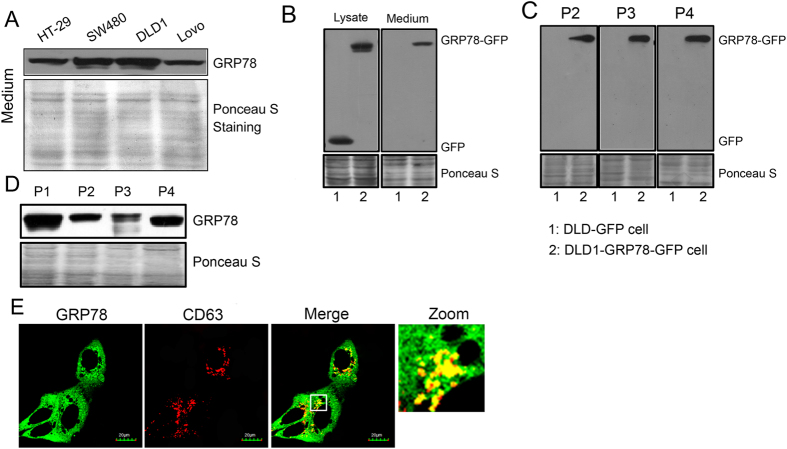
GRP78 is secreted from exosomes in colon cancer cells. (**A**) HT-29, SW480, DLD1 and Lovo cells were cultured in serum-free medium for 24 h. Supernatants were collected, centrifuged to remove cells, and subjected to SDS-PAGE followed by Western blotting with an anti-GRP78 antibody. (**B**) Immunoblots of GFP and GRP78-GFP in the whole-cell lysates and culture supernatants of DLD1 cells stably expressing GFP and GRP78-GFP. (**C**) Culture supernatants from DLD1 cells were subjected to differential centrifugation for 10 min at 300 × *g* (P1), 10 min at 2,000 × *g* (P2), 30 min at 10,000 × *g* (P3) and 3 h at 110,000 × *g* (P4). The P1–P4 pellets were analysed by immunoblotting for GRP78. (**D**) The culture supernatants from GRP78-GFP-expressing DLD1 cells were differentially centrifuged as described above, and the P2–P4 pellets were analysed by immunoblotting for GFP. (**E**) CD63 was immunostained in DLD1 cells stably expressing GRP78-GFP. The yellow dots correspond to foci where GRP78 (green) and CD63 (red) colocalized.

We previously demonstrated that GRP78 is secreted from colon cancer cells^17^. Here, we further demonstrate that among HT-29, SW480, DLD1 and Lovo colon cancer cell lines, DLD1 cells secrete a comparatively higher level of GRP78 (Fig. 1A). Notably, DLD1 cells expressing GFP-tagged GRP78 released the GRP78-GFP fusion protein extracellularly, whereas those cells expressing GFP did not release the GFP protein (Fig. 1B). To further confirm the secretory form of GRP78, culture supernatants from parental cells and cells expressing GRP78-GFP were fractionated by differential centrifugation, with P1, P2, P3 and P4 corresponding to floating cells, dead cells, cell debris and exosomes, respectively^19^. As shown in Fig. 1C,D, GRP78 and GRP78-GFP were present in all isolated fractions, including the exosome fraction. Interestingly, prominent colocalization between GRP78 and CD63, an intracellular exosome marker, was observed in the cytoplasm of DLD1 cells (Fig. 1E and Fig. S1). These observations indicate that GRP78 is selectively secreted via membrane vesicles.

### GRP78 secretion via membrane vesicles is reduced by HDAC inhibitors

**Figure 2:**
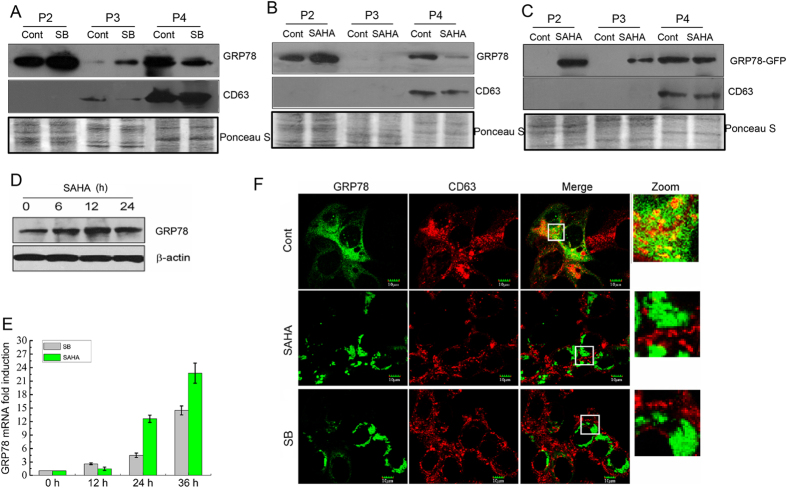
GRP78 secretion via exosomes is reduced by HDAC inhibitors. (**A,B**) Culture supernatants from DLD1 cells after sodium butyrate (SB) or SAHA treatment were subjected to differential centrifugation as described in Fig. 1. The P2–P4 pellets were analysed by immunoblotting for GRP78 and CD63. (**C**) Western blot detection of GFP in P2–P4 pellets obtained from the supernatants of DLD1 cells stably expressing GRP78-GFP with or without SAHA treatment. (**D**) Western blot detection of GRP78 and β-actin in whole-cell lysates of DLD1 cells after SAHA treatment for the indicated time intervals. (**E**) Relative mRNA levels of GRP78 in DLD1 cells at the indicated time points following SB or SAHA treatment. (**F**) DLD1 cells stably expressing GRP78-GFP were treated with SB or SAHA and immunostained with an anti-CD63 antibody. Superimposed confocal images demonstrate the colocalization of GRP78 and CD63.

We found that the membrane translocation of GRP78 was blocked by the HDAC inhibitor sodium butyrate^20^. To investigate whether pan-HDAC inhibitors can interfere with GRP78 secretion, parental and GRP78-GFP stably expressing DLD1 cells were treated with sodium butyrate (SB) or vorinostat (SAHA). Both treatments resulted in an increase in GRP78 and GRP78-GFP in the P2 and P3 fractions (Fig. 2A–C). Consistently, the global expression of GRP78 after SB and SAHA treatments was also markedly elevated at both the mRNA and protein levels (Fig. 2D,E). Strikingly, both inhibitors caused a dramatic reduction in GRP78 and GRP78-GFP in the P4 fraction (i.e., the exosome fraction, as evidenced by the presence of its characteristic protein CD63) (Fig. 2A–C). Furthermore, the frequently occurring colocalization of GRP78-GFP and CD63 within GRP78-GFP-expressing cells disappeared after SB or SAHA treatment (Fig. 2F). These results demonstrate that HDAC inhibitors inhibit the release of GRP78 via exosomes.

### HDAC inhibitors induce intracellular aggregation of GRP78 in the ER

**Figure 3:**
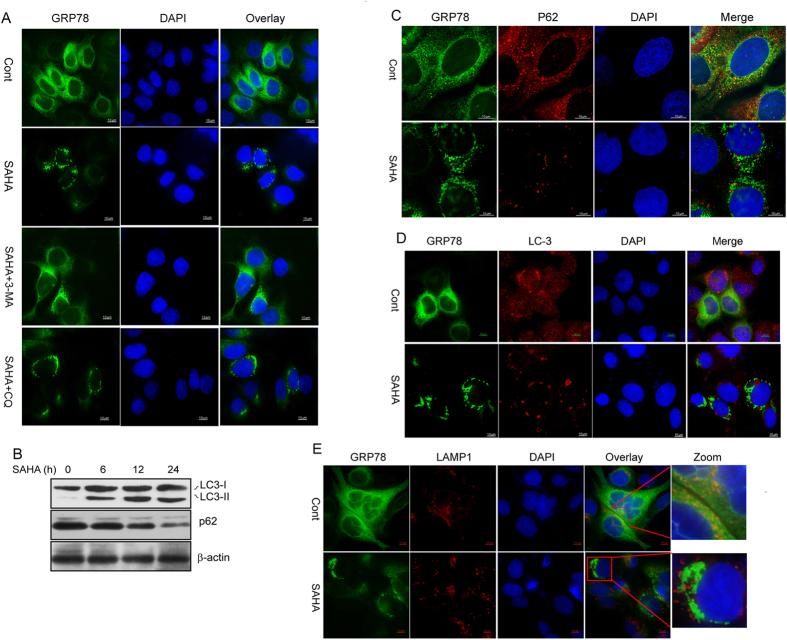
HDAC inhibitors induce GRP78 intracellular aggregation. (**A**) DLD1 cells stably expressing GRP78-GFP were treated with SAHA in the presence of 3-MA or CQ. The nucleus was stained with DAPI. (**B**) Western blot detection of LC-3I/II, p62 and β-actin in whole-cell lysates of DLD1 cells treated with SAHA for the indicated time intervals. (**C–E**) DLD1 cells stably expressing GRP78-GFP were treated with or without SAHA and immunostained with anti-p62, -LC-3 and -LAMP1 antibodies, respectively. The nucleus was stained with DAPI. The corresponding images were superimposed to determine the degrees of colocalization.

Interestingly, intracellular aggregation of GRP78 was readily observed after SB or SAHA treatment (Fig. 2F). We also found that SAHA treatment activated an autophagy response in DLD1 cells, as characterized by an increase in the LC3-II/I ratio and a decrease in p62 protein (Fig. 3A). Next, 3-methyl adenine (3-MA) and chloroquine (CQ), which impair Vps34/PIK3C3 activity and lysosomal degradation, respectively, were used to inhibit SAHA-induced autophagy. Notably, SAHA-induced GRP78 aggregation was almost completely abolished by 3-MA but not by CQ (Fig. 3B), suggesting that the GRP78 aggregation induced is likely to be related to cell autophagy. We then investigated the association of GRP78 aggregation with p62-positive protein aggregates, LC3-positive autophagosomes and LAMP1-positive lysosomes. As shown in Fig. 3C–E, no precise colocalization of GRP78 with p62, LC3 or LAMP1 was observed in DLD1 cells, regardless of SAHA treatment.

**Figure 4:**
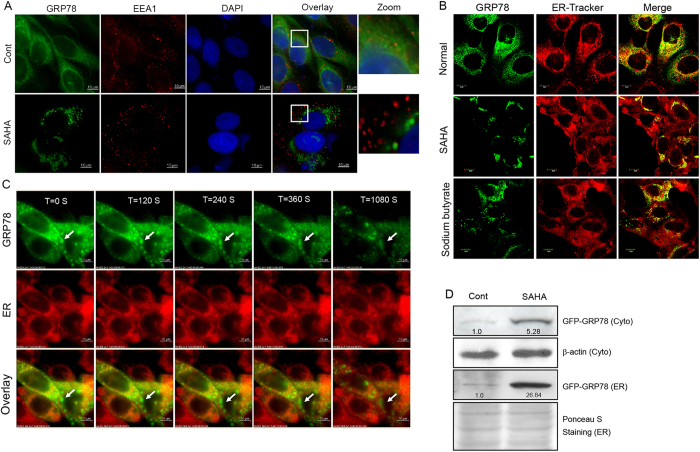
HDAC inhibitors induce GRP78 aggregation in the ER. (**A**) DLD1 cells stably expressing GRP78-GFP were treated with SAHA and immunostained with an anti-EEA1 antibody. The nucleus was stained with DAPI. The corresponding images were superimposed to determine the degrees of colocalization. (**B**) DLD1 cells stably expressing GRP78-GFP were treated with SAHA or sodium butyrate. The ER was labelled with ER-Tracker Red dye, and colocalization analysis was performed using confocal microscopy. (**C**) Immunofluorescence photomicrographs of GRP78-GFP-expressing DLD1 cells treated with SAHA at different time points (120, 240, 360, 1080 s). (**D**) ER fractions were isolated from GRP78-GFP-expressing cells with or without SAHA treatment for 24 h. After cell lysis, ER and cytoplasmic fractions were subjected to Western blot analysis to probe for GRP78-GFP.

Exosomes are released from an intracellular compartment, multivesicular bodies (MVBs), or late endosomes^21^. Given that MVBs may be derived from the ER or early endosomes (containing internalized membrane proteins)^22^ and that GRP78 is present in both the cell membrane and ER^13^, we hypothesize that the HDAC inhibitor-mediated decrease in GRP78 secretion via exosomes may be caused by its aggregation in the early endosome or ER. Although no colocalization signal between GRP78 and the early endosome marker EEA1 was observed before or after SAHA treatment (Fig. 4A), we did observe an apparent localization of GRP78 aggregates in the ER (Fig. 4B). Inspection under a DeltaVision microscope also demonstrated no positional changes during the aggregation process of GRP78 (Fig. 4C). To further corroborate the accumulation of GRP78 in the ER under SAHA treatment for 24 h, ER fractions were isolated from GRP78-GFP expressing cells with or without SAHA treatment; GRP78-GFP levels were induced by approximately 5-fold in the whole-cell lysate and approximately 27-fold in the ER fraction (Fig. 4D). These results indicate that HDAC inhibitor-induced GRP78 aggregation occurs *in situ* in the ER.

### GRP78 secretion is regulated by HDAC6

**Figure 5:**
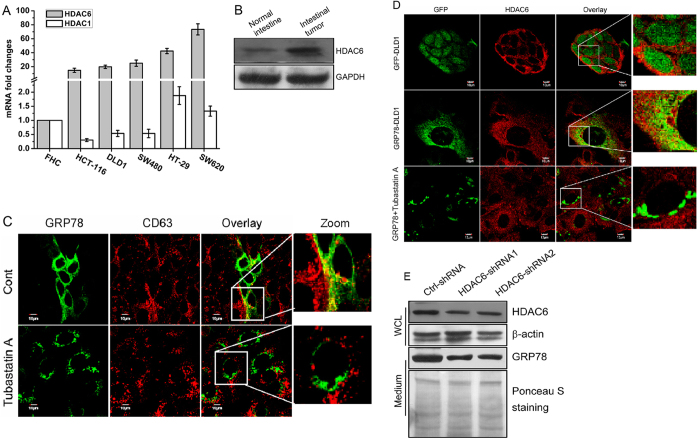
HDAC6 is implicated in regulating exosome-dependent GRP78 secretion. (**A**) Relative mRNA levels of HDAC1 and HDAC6 in normal colon FHC cells and colon cancer cells (HCT-116, DLD1, SW480, HT-29 and SW620). (**B**) Western blot analysis of HDAC6 in normal mouse intestine tissue or intestinal tumour tissue from an AOM/DSS-induced colon tumourigenesis model. (**C**) DLD1 cells stably expressing GRP78-GFP were treated with tubastatin A and immunostained with an anti-CD63 antibody. The superimposed confocal images show the colocalization of GRP78 and CD63. (**D**) DLD1 cells stably expressing GFP and GRP78-GFP were treated with tubastatin A and immunostained with an anti-HDAC6 antibody to analyse the relative intracellular location alterations of GRP78 and HDAC6. (**F**) HDAC6 expression in DLD1 cells was knocked down by shRNAs. Culture supernatants were collected and subjected to Western blot analysis to assess GRP78 secretion. Western blot analysis of HDAC6 expression was performed to confirm the knockdown efficiency of the shRNAs.

Considering that HDAC inhibitors can trigger GRP78 acetylation and that HDAC6 is involved in this modification^14^,^23^, we speculated that the decrease in GRP78 secretion may be associated with HDAC6-mediated acetylation. Compared with normal colon FHC cells, HDAC6, but not HDAC1, was highly expressed in all of the colon cancer cells examined (HCT-116, DLD1, SW480, HT-29 and SW620) (Fig. 5A). In addition, HDAC6 was also upregulated in the azoxymethane (AOM)+ DSS inflammation-driven colon tumourigenesis model (Fig. 5B). Inhibition of HDAC6 by its selective inhibitor tubastatin A resulted in GRP78 aggregation and the disappearance of GRP78-CD63 colocalization, reproducing the effects of SB and SAHA (Fig. 5C). Consistently, the colocalization signal between GRP78 and HDAC6 disappeared after tubastatin A treatment (Fig. 5D). Importantly, we demonstrated that HDAC6 depletion by shRNAs significantly suppressed GRP78 secretion (Fig. 5E). These results indicate that high HDAC6 expression at least partially accounts for a high level of secretion of GRP78 by cancer cells.

### GRP78 aggregation is associated with VPS34

**Figure 6:**
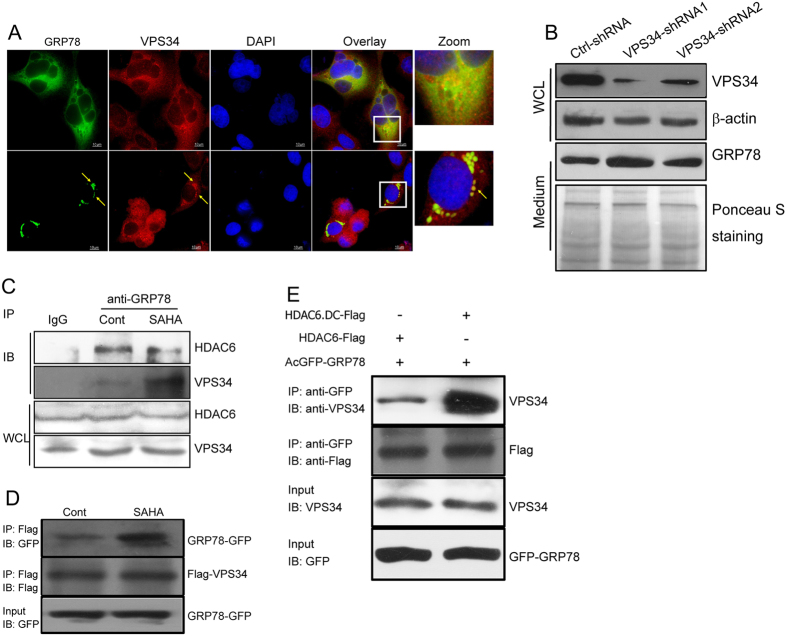
The aggregation of GRP78 is associated with VPS34. (**A**) DLD1 cells stably expressing GRP78-GFP were treated with or without SAHA and immunostained with an anti-VPS34 antibody. The images were superimposed to determine the degree of colocalization. (**B**) VPS34 expression in DLD1 cells was knocked down by shRNAs. Culture supernatants were collected and subjected to Western blot analysis of GRP78 secretion. (**C**) DLD1 cells treated or untreated with SAHA were immunoprecipitated with an anti-GRP78 antibody followed by immunoblotting with anti-HDAC6 and anti-VPS34 antibodies. (**D**) 293T cells were transfected with Flag-tagged VPS34 and GRP78-GFP plasmids. After transfection for 24 h, cells were treated with SAHA for another 12 h. Lysates from SAHA-treated and untreated cells were immunoprecipitated with an anti-Flag antibody followed by immunoblotting with anti-GFP and anti-Flag antibodies. (**E**) 293T cells were co-transfected with GRP78-GFP and Flag-tagged wild-type or catalytically inactive HDAC6. Cell lysates were immunoprecipitated with an anti-GFP antibody followed by immunoblotting with anti-VPS34 and anti-Flag antibodies.

Given that HDAC inhibitor-triggered GRP78 aggregation can be abrogated by 3-MA, an inhibitor of VPS34/Class III PI3K, and that GRP78 can interact with Class I PI3K, the catalytic subunit of which exhibits considerable homology with VPS34^24^,^25^, we hypothesized that HDAC inhibitor-induced GRP78 aggregation may be associated with VPS34. Indeed, we observed an evident colocalization of GRP78 with VPS34, which became much stronger after SAHA treatment (Fig. 6A), and VPS34-knockdown cells released a greater amount of GRP78 into the medium in the presence of SAHA (Fig. 6B). We next compared the changes in the GRP78-VPS34 interection with and without SAHA treatment by a Co-IP assay. As shown in Fig. 6C, the GRP78-VPS34 interaction was enhanced upon SAHA stimulation, whereas the GRP78-HDAC6 interaction was appreciably compromised. Increased interaction between GRP78 and VPS34 after SAHA stimulation was also observed in 293T cells transfected with GRP78-GFP- and Flag-VPS34-expressing plasmids (Fig. 6D). Notably, inactivation of HDAC6 via transfection of its catalytically inactive mutant also significantly augmented the GRP78-VPS34 interaction (Fig. 6E). Together, these results indicate that HDAC inhibitor treatment results in increased interaction between GRP78 and VPS34 and that HDAC6 is involved in regulating this interaction.

### GRP78 acetylation affects its secretion and tumour growth

**Figure 7:**
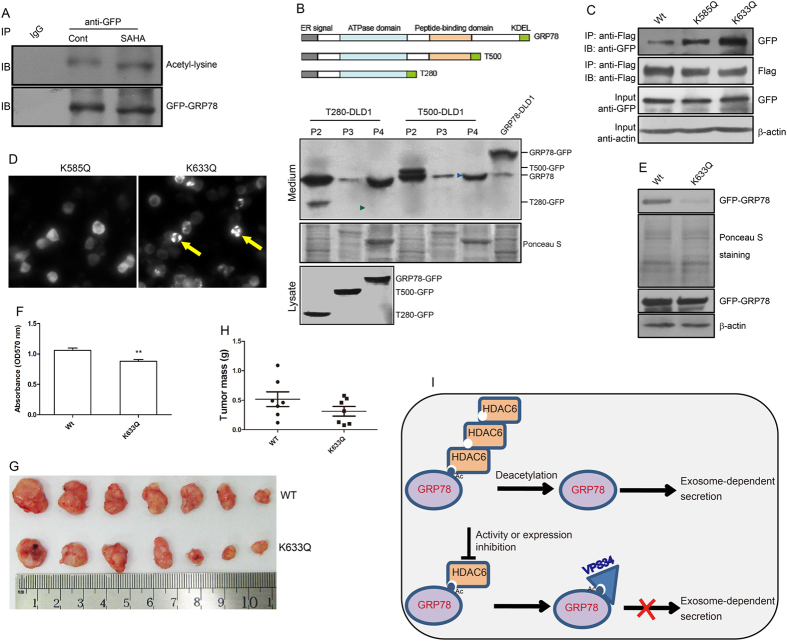
GRP78 acetylation affects its secretion and tumour growth. (**A**) Culture supernatants from DLD1 cells stably expressing wild-type GRP78 or deletion mutants were collected and subjected to differential centrifugation, as described in Fig. 1. The P2–P4 pellets were analysed by immunoblotting for GFP. (**B**) Cell lysates from GRP78-GFP-expressing cells treated with or without SAHA were immunoprecipitated with an anti-GFP antibody, and an immunoblot was performed with an anti-acetyl-lysine antibody. (**C**) Wild-type GRP78, the K585Q mutant or the K633Q mutant was co-transfected with the VPS34-Flag plasmid into 293T cells, and lysates were immunoprecipitated with an anti-Flag antibody followed by immunoblotting with an anti-GFP antibody. Ten percent of the total cell lysate used in immunoprecipitation was used as the input. (**D**) The K585Q and K633Q mutants were transfected into 293T cells, and the distribution of green fluorescence was observed by fluorescence microscopy. (**E**) Culture supernatants from DLD1 cells expressing wild-type GRP78 and the K633Q mutant were collected and subjected to an analysis of GRP78 secretion by Western blotting. (**F**) An MTT assay was used to assess the growth of cells expressing wild-type GRP78 and the K633Q mutant. (**G,H**) Equal numbers of wild-type GRP78- and K633Q mutant-expressing cells were injected into immunocompromised mice. The dissected tumours, in pairs, and the bar graph representing the tumour weight are shown. (**I**) A schematic representation of the proposed model. In tumour cells highly expressing HDAC6, GRP78 is deacetylated and enters an exosome-dependent secretory pathway; in cells expressing low amounts of HDAC6 or upon inhibition by HDAC inhibitors, GRP78 cannot be deacetylated by HDAC6. Acetylated GRP78 is thus recruited to the VPS34 complex, preventing GRP78 sorting into the exosomes.

The results presented above suggest that GRP78 secretion by colon cancer cells is likely associated with its acetylation status. Consistent with this hypothesis, GRP78-GFP acetylation was significantly elevated in response to SAHA treatment (Fig. 7A). To determine the functional domains of GRP78 required for its secretion, supernatants from cells stably expressing wild-type GRP78 or deletion mutants were collected and subjected to differential centrifugation and Western blot analysis. Neither of the GRP78 mutants, which lack a C-terminus (~150 amino acids), were released extracellularly via exosomes (Fig. 7B), suggesting that this amino acid sequence likely plays an important role in GRP78 secretion.

It has been reported that GRP78 can be acetylated at K118, K122, K123, K125, K138, K152, K154, K353, K353, K376, K585 and K633^14^,^23^. Because the K585 and K633 sites are located within the C-terminal region, these sites were mutated to glutamine (Q) to mimic the acetylated form of lysine and named K585Q and K633Q, respectively. Interaction of the K633Q mutant with Flag-VPS34 became more intense than that of the wild type (WT, Fig. 7C). In contrast to the K585Q mutant, we observed an obvious aggregation of the K633Q mutant in transfected 293T cells, mimicking the effects of SAHA stimulation (Fig. 7D). Importantly, the K633Q mutation also resulted in decreased GRP78 secretion (Fig. 7E). We next asked whether GRP78 K633 acetylation affects tumour cell growth. To address this question, DLD1 cells were stably infected with lentivirus expressing wild-type GRP78 and the K633Q mutant. As shown in Fig. 7F, an MTT assay revealed significant growth impairment for the K633Q mutant-expressing cells. We then injected cells into immunocompromised (nu/nu) mice and assessed tumour formation. Tumours derived from the K633Q mutant-expressing cells were smaller compared with those expressing the wild-type GRP78, though no statistically significant difference was found between these two groups (Fig. 7G,H).

## Discussion

There is emerging evidence that tumour and tumour stromal cells release exosomes that participate in local and systemic cell communication in an autocrine/paracrine manner. Exosomes have a topology similar to cells and contain a wide range of biologically active materials, including cytokines, growth factors, extracellular matrix molecules, mRNAs and microRNAs^21^. Exosomes containing RNA molecules may act as a vehicle for the horizontal transfer of genetic information between cells^7^. HSP70 and HSP90α, both of which are classical cytosolic chaperones, have been reported to be secreted in the form of exosomes^26^,^27^. In this study, we demonstrate that the ER chaperone GRP78 can also be released into the extracellular space via exosomes. Given that GRP78 exhibits diverse cellular distribution in cancer cells, there may be at least three routes by which GRP78 enters MVBs, including ER, plasma membrane and cytoplasmic routes. As our data do not show an obvious localization of GRP78 in the early endosome, we can exclude the plasma membrane-endosome-exosome pathway. Nonetheless, the specific sorting route of GRP78 into exosomes remains unclear, and further investigation is warranted.

Protein acetylation controlled by the balance between the activities of histone acetyltransferases (HATs) and histone deacetylases (HDACs) plays a key role in transcriptional regulation in the nucleus. In this study, an increased endogenous GRP78 gene expression was induced following treatment with the HDAC inhibitor SAHA. This is most likely due to the activation of GRP78 promoter by SAHA, as evidenced by Kia A *et al*. in a viral vector^28^. Recently, non-nuclear protein acetylation and its role in cellular regulation, especially in cell metabolism and autophagy, have attracted great attention^29^,^30^,^31^,^32^,^33^. HDAC6, a cytoplasmic class II HDAC, functions in many cellular events by deacetylating non-histone proteins, including HSP90 and GRP78^14^,^34^. Upon HDAC6 inhibition by panobinostat, GRP78 is acetylated at 11 lysine residues, resulting in its dissociation from PERK and activation of a lethal unfolded protein response (UPR) in breast cancer cells^14^. Interestingly, we demonstrate that HDAC6 is highly expressed in colon cancer cells. Inhibition of HDAC6 activity by two pan-HDAC inhibitors and an HDAC6 selective inhibitor provokes GRP78 aggregation in the ER and its sorting into MVBs, culminating in a decrease in GRP78 secretion via exosomes. We further identify that blocking GRP78 release by inhibiting HDAC6 is largely attributable to acetylation of GRP78 at K633. Thus, there are at least two reasons accounting for the retard of tumour growth induced by GRP78 acetytion: activation of a lethal UPR and blockage of GRP78 secretion.

How does acetylation affect the sorting of GRP78 into exosomes? Strikingly, we observed that GRP78 acetylation leads to a decrease in its affinity toward HDAC6 but an increase in interaction with VPS34, a class III phosphatidylinositol-3 kinase that can form distinct complexes involved in vesicular transport or autophagy^35^,^36^. Yang *et al*. reported that autophagy-inducing stresses (including HDAC inhibitor treatment) increase intracellular levels of acetylated HSP70, which binds to the Vps34-Beclin-1 complex to promote autophagic vesicle formation^37^; interestingly, GRP78 was also detected in the panobinostat-induced Vps34 complex by mass spectrometry assays^37^. Thus, the most likely scenario appears to be that the C-terminus of GRP78 serves as a common platform for interacting with both HDAC6 and VPS34. Upon HDAC6 inhibition, GRP78 is acetylated and recruited to the VPS34 complex, which facilitates VPS34-mediated autophagy. By extension, this autophagy response may also be responsible for chemotherapeutic resistance to HDAC inhibitors.

Cancer progression depends on the interaction between tumour cells and the tumour microenvironment, which includes extracellular matrix components, various stromal cells and specific growth conditions characterized by hypoxia, glucose deficiency and lactic acidosis^38^. The exosome is identified as an efficacious signal mediator between tumour cells and tumour stromal cells, and recent work indicates that the release and function of exosomes are intertwined with the conditions of the extracellular environment, such as hypoxia and an acidic pH^11^,^39^. GRP78 is a centrally located sensor of stress and is usually highly induced by glucose deprivation, hypoxia and acidosis. Thus, it is reasonable to propose that on the one hand, GRP78 is frequently highly expressed due to the persistent microenvironmental stress present in solid tumours; a high level of HDAC6 is responsible for the comparatively low acetylated status of GRP78, preventing GRP78 aggregation and allowing it to be sorted into exosomes. On the other hand, the acidic pH of the microenvironment accelerates the release of exosomes containing GRP78, which potently promotes tumour progression in an autocrine/paracrine manner. Taken together, this work provides insight into how GRP78 acetylation affects its secretion via exosomes in colon cancer, and the findings have therapeutic implications for the use of HDAC inhibitors in cancer therapy.

## Methods

### Materials

RPMI-1640 and DMEM medium and foetal bovine serum (FBS) were obtained from GIBCO (Grand Island, NY). Trizol, PrimeScript RT Master Mix and SYBR green PCR master mix were purchased from Takara (Shiga, Japan). Antibodies against LC-3I/II, p62, VPS34, and HDAC6 were purchased from Cell Signaling Technology (Danvers, USA), antibodies against GFP, GRP78 and acetyl-lysine were from Abcam (Cambridge, UK), and anitbodies against β-tubulin, β-actin and GAPDH were from Abmart (Shanghai, China). The anti-CD63 antibody was purchased from Santa Cruz Biotechnology (Santa Cruz, USA), and the anti-FITC-, -TRITC-, -Cy5- and -HRP-conjugated secondary antibodies as well as ER-Tracker and Lyso-Tracker were obtained from Invitrogen (Carlsbad, USA). Sodium butyrate, vorinostat (SAHA), tubastatin A and 3-methyladenine were obtained from Cayman Chemical (MI, USA). Anti-FLAG M2 mAb and anti-FLAG M2 agarose were obtained from Sigma (St. Louis, USA).

### Cell culture, HDAC inhibitor treatment and cell number determination

Human colon carcinoma cell lines HT-29, HCT-116, SW480, SW620 and DLD1 were cultured in RPMI-1640 medium containing 10% FBS at 37 °C and in 5% CO_2_ in a humidified incubator. Sodium butyrate (2 mM), SAHA (5 μM), tubastatin A (5 μM), 3-methyl adenine (3-MA, 10 mM) and chloroquine (25 μM) were applied to cell cultures when necessary. Cell viability was assessed by the MTT assay, as described elsewhere^40^.

### Cell culture supernatant, exosome and ER isolation

For cell culture supernatants collection, cells were cultured in DMEM and incubated for 24 h. The medium was collected and centrifuged at 2,000 × g for 10 min. The supernatants were concentrated with a 10 kDa ultrafiltration membranes (Millipore, MA). For exosome isolation, cells were cultured in serum-free DMEM medium for 24 h. The culture supernatants were subjected to differential centrifugation for 10 min at 300 × *g* (P1), 10 min at 2,000 × *g* (P2), 30 min at 10,000 × *g* (P3) and 3 h at 110,000 × *g* (P4, exosome pellets)^19^, and different pellets were washed in PBS and lysed with in Western and IP buffer (Beyotime, China) containing a protease inhibitor cocktail (Thermo scientific). The protein concentration was measured with the BCA Protein Assay Reagent (Beyotime, China). Samples with equal protein loading (30 ~ 50 μg) were subjected to Western blot analysis. For ER isolation, an ER isolation kit (Sigma) was used according to the manufacturer’s instructions. Briefly, after SAHA treatment for 24 h, 5 × 10^8^ cells were harvested, resuspended in hypotonic buffer at a volume three times that of the packed pellet, and mechanically disrupted using a Dounce homogenizer. Cell extracts were then subjected to a discontinuous iodixanol gradient by centrifugation at 100,000 × g for 16 h. ER Fractions were collected, solubilized in Western and IP buffer and and analysed by Western blotting.

### Truncated and site mutant construction

DNA fragments encoding different truncated GRP78 mutants were amplified from the plasmid PLVX-GRP78-AcGFP, which was previously constructed in our lab^41^. Primers (T500: 5′-CCGCTCGAGATGAAGCTCTCCCTGGTGGCCGC-3′, 5′-CGCGGATCCCGCAACTCATCTTTGGTGACTTCAATCTGTGG-3′; T280: CCGCTCGAGATGAAGCTCTCCCTGGTGGCCGC, CGCGGATCCCGCAACTCATCTTTTTTCCTGACATCTTTGCC) introducing XhoI and BamHI restriction sites were used to obtain the target fragments, which were then cloned into pLVX-AcGFP1-N1 (Clontech). Successful cloning was confirmed by sequencing. The PLVX-GRP78-AcGFP-K585Q and -K633Q mutants were constructed using Easy Mutagenesis System from Transgen Biotech (Beijing, China).

### Cell transfection and lentivirus infection

For transient transfection, plasmids were introduced into 293T cells using the transfection reagent TurboFect (Thermo Scientific). For lentivirus infection, the overexpression/shRNA plasmids PsPax2 and pMD2.G were co-transfected into 293T cells using the calcium phosphate method at 15: 12.5: 3.75 g (for a 10-cm dish). The virus-containing medium was collected at 48 h after transfection and was then concentrated using 100-kDa ultrafiltration membranes (Millipore). Cells were infected with the viruses in the presence of polybrene (8 μg/ml) for 48 h and then subjected to selection with 5 μg/ml puromycin for 72 hours.

### RNA extraction and real-time PCR analysis

Total RNA extraction, reverse transcription, and real-time PCR were performed as previously described^20^. The primers used in this study were as follows: GRP78: 5′-CTGTGCAGCAGGACATCAAGTTC-3′; 5′-TGTTTGCCCACCTCCAATATCA-3′. HDAC6: 5′-GGATGTGCACCACGGTCAAG-3′; 5′-GAAACCTGTGGTGGACCAGTTAGAG-3′. HDAC1: 5′-AGTATTCGATGGCCTGTTTGAGTTC-3′; 5′-CAGTTCCAGGATGGCCAAGA-3′. GAPDH: 5′-GCACCGTCAAGGCTGAGAAC-3′; 5′-TGGTGAAGACGCCAGTGGA-3′.

### Immunofluorescence

Cells were seeded onto 6-well glass slides. After treatment, the cells were fixed in 4% paraformaldehyde in PBS for 30 min and permeabilized with 0.3% Triton X-100 in PBS for 10 min. Next, the slides were blocked in 2% goat serum for 1 h and incubated with primary antibodies at 4 °C overnight. The slides were then washed and incubated with the corresponding secondary antibodies. After three PBS washes, the slides were mounted in gelvatol for confocal immunofluorescence analysis.

### Co-immunoprecipitation and Western blot assays

Cells were lysed in Western and IP buffer (Beyotime, China) containing a protease inhibitor cocktail (Thermo scientific). 500 μg protein of whole-cell lysates (WCLs) with a final volume of 1 ml were pre-cleared by incubation with 1.0 μg control IgG corresponding to the host species of the primary antibody together with 20 μl of Protein A/G PLUS-Agarose (Santa Cruz) at 4 °C for 2 h followed by centrifugation at 4 °C for 5 min at 2,500 rpm. The supernatant was subjected to immunoprecipitation by the addition of 2 μg of immunoprecipitation antibodies and incubated overnight at 4 °C followed by incubation with Protein A/G PLUS-Agarose for 2 h. After washing with cell lysis buffer four times, the beads were boiled in 2 × SDS loading buffer, and the supernatants were resolved by SDS-PAGE and subjected to Western blot analysis, as previously described^20^.

### Xenograft tumour assay

All of the mouse experiments were approved by the Institutional Animal Care and Use Committee of Shanxi University (Taiyuan, China). All of the experimental procedures were performed in accordance with the protocols and ethical regulations approved by the Institutional Animal Care and Use Committee of Shanxi University (Taiyuan, China). Four-week-old female nude mice were used for this study. Equal amounts (5 × 10^6^) of DLD1 cells expressing the K585Q or K633Q mutant were resuspended in 200 μl of PBS buffer and injected into the subcutis of immunocompromised (nu/nu) mice. Four weeks after injection, the mice were euthanized, and the tumours were excised and weighed.

### Statistical analysis

Data are expressed as the mean ± SEM. Differences among groups were tested by a one-way analysis of variance (ANOVA), and comparisons between two groups were evaluated using Student’s t-test. A value of p < 0.05 was considered statistically significant.

## Additional Information

**How to cite this article**: Li, Z. *et al*. Acetylation modification regulates GRP78 secretion in colon cancer cells. *Sci. Rep.* **6**, 30406; doi: 10.1038/srep30406 (2016).

## Supplementary Material

Supplementary Information

## References

[b1] Grieve A. G. & Rabouille C. Golgi Bypass: Skirting Around the Heart of Classical Secretion. Csh Perspect Biol 3, 1–15 (2011) 10.1101/cshperspect.a005298 PMC3062214 21441587

[b2] Muralidharan-Chari V., Clancy J. W., Sedgwick A. & D’Souza-Schorey C. Microvesicles: mediators of extracellular communication during cancer progression. J Cell Sci 123, 1603–1611 (2010). 20445011 10.1242/jcs.064386 PMC2864708

[b3] Simons M. & Raposo G. Exosomes - vesicular carriers for intercellular communication. Curr Opin Cell Biol 21, 575–581 (2009). 19442504 10.1016/j.ceb.2009.03.007

[b4] Raposo G. & Stoorvogel W. Extracellular vesicles: Exosomes, microvesicles, and friends. J Cell Biol 200, 373–383 (2013). 23420871 10.1083/jcb.201211138 PMC3575529

[b5] Colombo M., Raposo G. & Thery C. Biogenesis, Secretion, and Intercellular Interactions of Exosomes and Other Extracellular Vesicles. Annu Rev Cell Dev Bi 30, 255–289 (2014). 10.1146/annurev-cellbio-101512-122326 25288114

[b6] Thery C., Ostrowski M. & Segura E. Membrane vesicles as conveyors of immune responses. Nat Rev Immunol 9, 581–593 (2009). 19498381 10.1038/nri2567

[b7] Skog J. . Glioblastoma microvesicles transport RNA and proteins that promote tumour growth and provide diagnostic biomarkers. Nat Cell Biol 10, 1470–U1209 (2008). 19011622 10.1038/ncb1800 PMC3423894

[b8] Luga V. . Exosomes Mediate Stromal Mobilization of Autocrine Wnt-PCP Signaling in Breast Cancer Cell Migration. Cell 151, 1542–1556 (2012). 23260141 10.1016/j.cell.2012.11.024

[b9] Peinado H. . Melanoma exosomes educate bone marrow progenitor cells toward a pro-metastatic phenotype through MET. Nat Med 18, 883–891 (2012). 22635005 10.1038/nm.2753 PMC3645291

[b10] Kahlert C. & Kalluri R. Exosomes in tumor microenvironment influence cancer progression and metastasis. J Mol Med 91, 431–437 (2013). 23519402 10.1007/s00109-013-1020-6 PMC4073669

[b11] Kucharzewska P. . Exosomes reflect the hypoxic status of glioma cells and mediate hypoxia-dependent activation of vascular cells during tumor development. P Natl Acad Sci USA 110, 7312–7317 (2013). 10.1073/pnas.1220998110 PMC3645587 23589885

[b12] Lee A. S. GRP78 induction in cancer: Therapeutic and prognostic implications. Cancer Res 67, 3496–3499 (2007). 17440054 10.1158/0008-5472.CAN-07-0325

[b13] Li Z. W. & Li Z. Y. Glucose regulated protein 78: A critical link between tumor microenvironment and cancer hallmarks. Bba-Rev Cancer 1826, 13–22 (2012). 10.1016/j.bbcan.2012.02.001 22426159

[b14] Rao R. . Treatment with Panobinostat Induces Glucose-Regulated Protein 78 Acetylation and Endoplasmic Reticulum Stress in Breast Cancer Cells. Mol Cancer Ther 9, 942–952 (2010). 20371724 10.1158/1535-7163.MCT-09-0988

[b15] Ni M., Zhang Y. & Lee A. S. Beyond the endoplasmic reticulum: atypical GRP78 in cell viability, signalling and therapeutic targeting. Biochem J 434, 181–188 (2011). 21309747 10.1042/BJ20101569 PMC3353658

[b16] Kern J. . GRP-78 secreted by tumor cells blocks the antiangiogenic activity of bortezomib. Blood 114, 3960–3967 (2009). 19713465 10.1182/blood-2009-03-209668

[b17] Peng Y. A., Li Z. W. & Li Z. Y. GRP78 secreted by tumor cells stimulates differentiation of bone marrow mesenchymal stem cells to cancer-associated fibroblasts. Biochem Bioph Res Co 440, 558–563 (2013). 10.1016/j.bbrc.2013.09.108 24113381

[b18] Fu R., Yang P., Wu H. L., Li Z. W. & Li Z. Y. GRP78 Secreted by Colon Cancer Cells Facilitates Cell Proliferation via PI3K/Akt Signaling. Asian Pac J Cancer P 15, 7245–7249 (2014). 10.7314/apjcp.2014.15.17.7245 25227822

[b19] Gross J. C., Chaudhary V., Bartscherer K. & Boutros M. Active Wnt proteins are secreted on exosomes. Nat Cell Biol 14, 1036–1045 (2012). 22983114 10.1038/ncb2574

[b20] Li Z. W. . Cell-surface GRP78 facilitates colorectal cancer cell migration and invasion. Int J Biochem Cell B 45, 987–994 (2013). 10.1016/j.biocel.2013.02.002 23485528

[b21] Record M., Subra C., Silvente-Poirot S. & Poirot M. Exosomes as intercellular signalosomes and pharmacological effectors. Biochem Pharmacol 81, 1171–1182 (2011). 21371441 10.1016/j.bcp.2011.02.011

[b22] Fevrier B. & Raposo G. Exosomes: endosomal-derived vesicles shipping extracellular messages. Curr Opin Cell Biol 16, 415–421 (2004). 15261674 10.1016/j.ceb.2004.06.003

[b23] Kahali S. . Activation of the Unfolded Protein Response Contributes toward the Antitumor Activity of Vorinostat. Neoplasia 12, 80–86 (2010). 20072656 10.1593/neo.91422 PMC2805886

[b24] Backer J. M. The regulation and function of Class III PI3Ks: novel roles for Vps34. Biochem J 410, 1–17 (2008). 18215151 10.1042/BJ20071427

[b25] Zhang Y. . Cancer Cells Resistant to Therapy Promote Cell Surface Relocalization of GRP78 Which Complexes with PI3K and Enhances PI(3,4,5)P3 Production. Plos One 8, e80071 (2013). 24244613 10.1371/journal.pone.0080071 PMC3823711

[b26] Gastpar R. . Heat shock protein 70 surface-positive tumor exosomes stimulate migratory and cytolytic activity of natural killer cells. Cancer Res 65, 5238–5247 (2005). 15958569 10.1158/0008-5472.CAN-04-3804 PMC1785299

[b27] McCready J., Sims J. D., Chan D. & Jay D. G. Secretion of extracellular hsp90 alpha via exosomes increases cancer cell motility: a role for plasminogen activation. Bmc Cancer 10, 294–303 (2010). 20553606 10.1186/1471-2407-10-294 PMC3087318

[b28] Kia A., Yata T., Hajji N. & Hajitou A. Inhibition of histone deacetylation and DNA methylation improves gene expression mediated by the adeno-associated virus/phage in cancer cells. Viruses 5, 2561–2572, 10.3390/v5102561 (2013). 24153059 PMC3814604

[b29] Shin J. Y., Zhang D. & Chen D. Reversible Acetylation of Metabolic Enzymes Celebration: SIRT2 and p300 Join the Party. Mol Cell 43, 3–5 (2011). 21726804 10.1016/j.molcel.2011.06.010

[b30] Wang Q. J. . Acetylation of Metabolic Enzymes Coordinates Carbon Source Utilization and Metabolic Flux. Science 327, 1004–1007 (2010). 20167787 10.1126/science.1179687 PMC4183141

[b31] Zhao S. M. . Regulation of Cellular Metabolism by Protein Lysine Acetylation. Science 327, 1000–1004 (2010). 20167786 10.1126/science.1179689 PMC3232675

[b32] Xu W., Li Y., Liu C. & Zhao S. Protein lysine acetylation guards metabolic homeostasis to fight against cancer. Oncogene 33, 2279–2285 (2014). 23665675 10.1038/onc.2013.163

[b33] Banreti A., Sass M. & Graba Y. The emerging role of acetylation in the regulation of autophagy. Autophagy 9, 819–829 (2013). 23466676 10.4161/auto.23908 PMC3672293

[b34] Yang Y. H. . Role of acetylation and extracellular location of heat shock protein 90 alpha in tumor cell invasion. Cancer Res 68, 4833–4842 (2008). 18559531 10.1158/0008-5472.CAN-08-0644 PMC2665713

[b35] Kim J. . Differential Regulation of Distinct Vps34 Complexes by AMPK in Nutrient Stress and Autophagy. Cell 152, 290–303, 10.1016/j.cell.2012.12.016 (2013). 23332761 PMC3587159

[b36] Marsh T. & Debnath J. Ironing out VPS34 inhibition. Nat Cell Biol 17, 1–3, 10.1038/ncb3089 (2015). 25679028

[b37] Yang Y. H. . Acetylated hsp70 and KAP1-mediated Vps34 SUMOylation is required for autophagosome creation in autophagy. P Natl Acad Sci USA 110, 6841–6846 (2013). 10.1073/pnas.1217692110 PMC3637746 23569248

[b38] Swartz M. A. . Tumor Microenvironment Complexity: Emerging Roles in Cancer Therapy. Cancer Res 72, 2473–2480 (2012). 22414581 10.1158/0008-5472.CAN-12-0122 PMC3653596

[b39] Parolini I. . Microenvironmental pH Is a Key Factor for Exosome Traffic in Tumor Cells. J Biol Chem 284, 34211–34222 (2009). 19801663 10.1074/jbc.M109.041152 PMC2797191

[b40] Chen J. . Specific receptor subtype mediation of LPA-induced dual effects in cardiac fibroblasts. FEBS Lett 580, 4737–4745 (2006). 16890224 10.1016/j.febslet.2006.07.061

[b41] Li Z. W. . GRP78 enhances the glutamine metabolism to support cell survival from glucose deficiency by modulating the beta-catenin signaling. Oncotarget 5, 5369–5380 (2014). 24977433 10.18632/oncotarget.2105 PMC4170599

