# Supplementary material for: Acetylation modification regulates GRP78 secretion in colon cancer cells
Source: Sci Rep. 2016 Jul 27;6:30406. doi: 10.1038/srep30406 (PMC4961953; doi:10.1038/srep30406)

Supplementary Information

# Acetylation modification regulates GRP78 secretion in colon cancer cells

Zongwei Li[1,3#], Ming Zhuang[2#], Lichao Zhang[1], Xingnan Zheng[4], Peng Yang[1], Zhuoyu Li[1*]

[1]Institute of Biotechnology, Key Laboratory of Chemical Biology and Molecular Engineering of National Ministry of Education, Shanxi University, Taiyuan, 030006, China.

[2]General Surgical Department, Xinhua Hospital Affiliated to Shanghai Jiao Tong University School of Medicine, Shanghai, 200092, China

[3]Department of Lymphoma and Myeloma, Division of Cancer Medicine, Center for Cancer Immunology Research, The University of Texas MD Anderson Cancer Center, Houston, Texas, USA

[4]Lineberger Comprehensive Cancer Center, University of North Carolina School of Medicine, Chapel Hill, North Carolina 27599, USA

#The first two authors contributed equally to this work.

***Corresponding Author: Zhuoyu Li**

Institute of Biotechnology, Key Laboratory of Chemical Biology and Molecular Engineering of National Ministry of Education, Shanxi University, Taiyuan 030006, China

Tel: 86-351-7018268, Fax: 86-351-7018268, Email: lzy@sxu.edu.cn.

Fig.1A

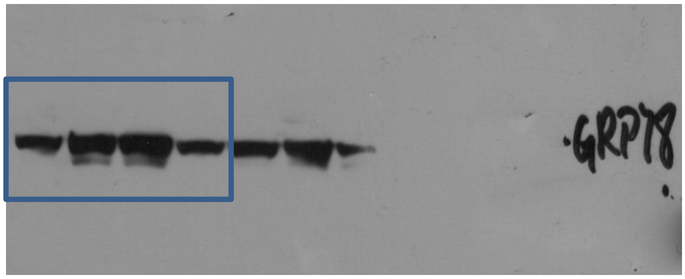


Fig.1B

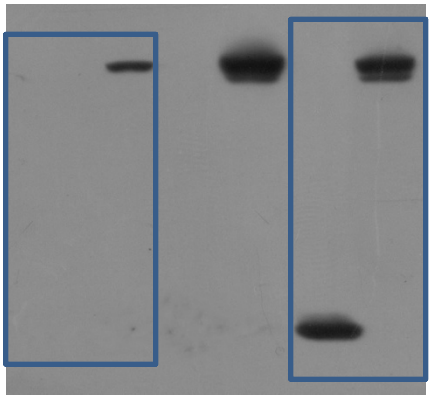

Fig.1C

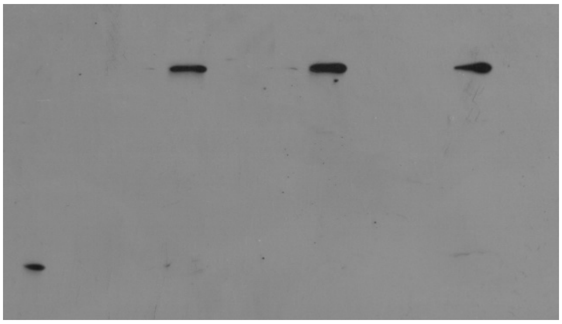

Fig. 1D

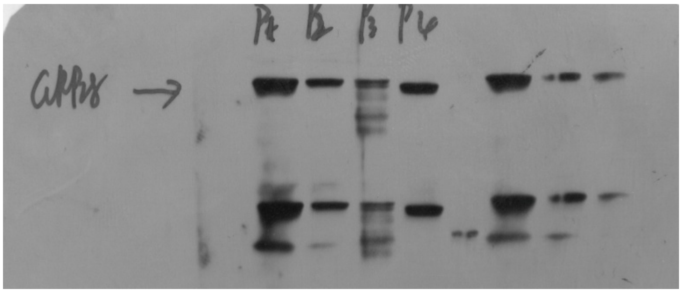

Fig.2A

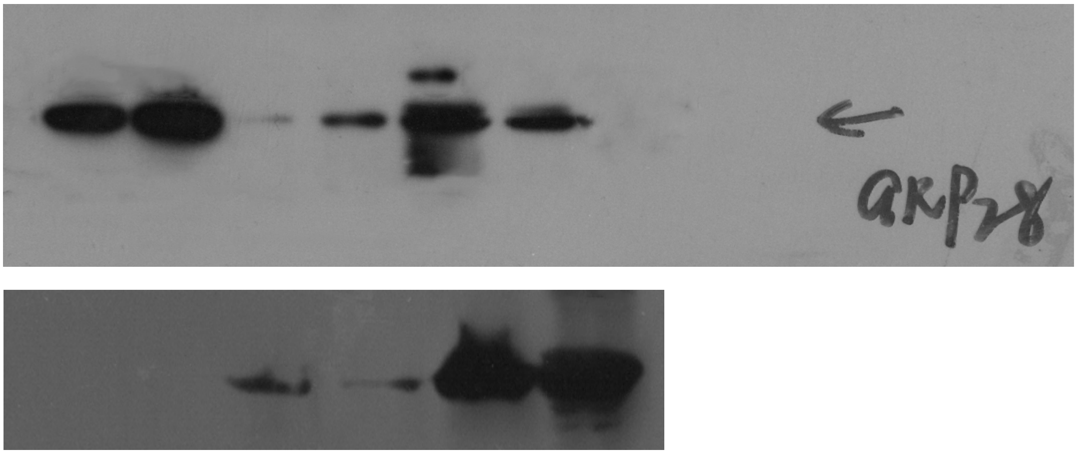

Fig.2B

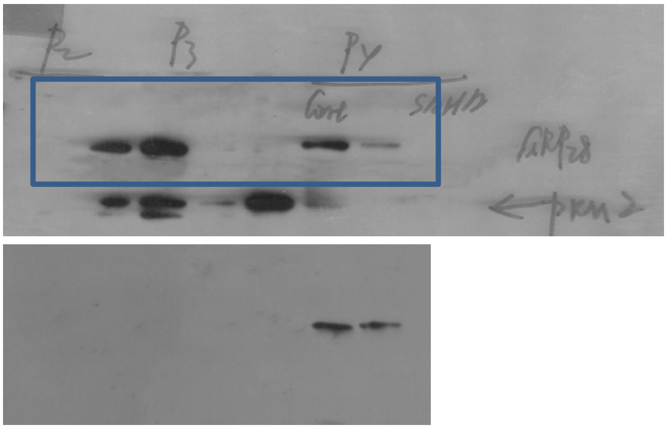

Fig.2C

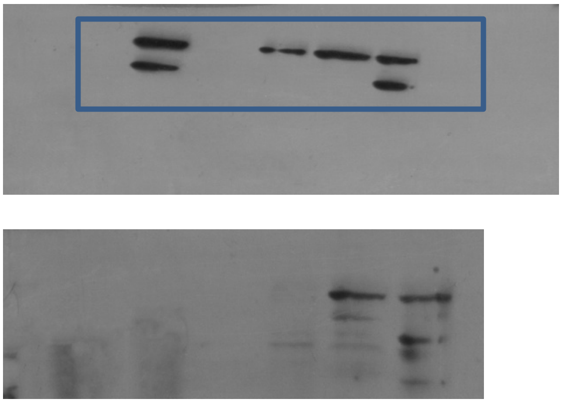

Fig.2D

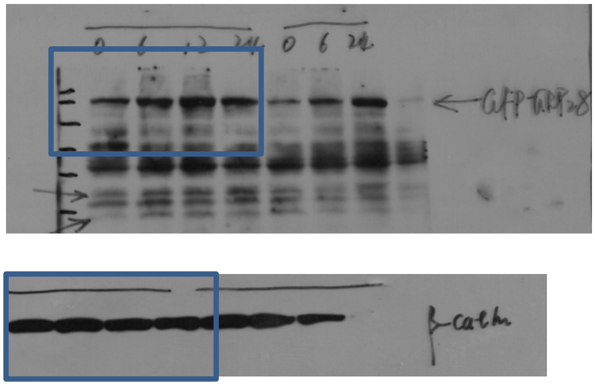

Fig. 3B

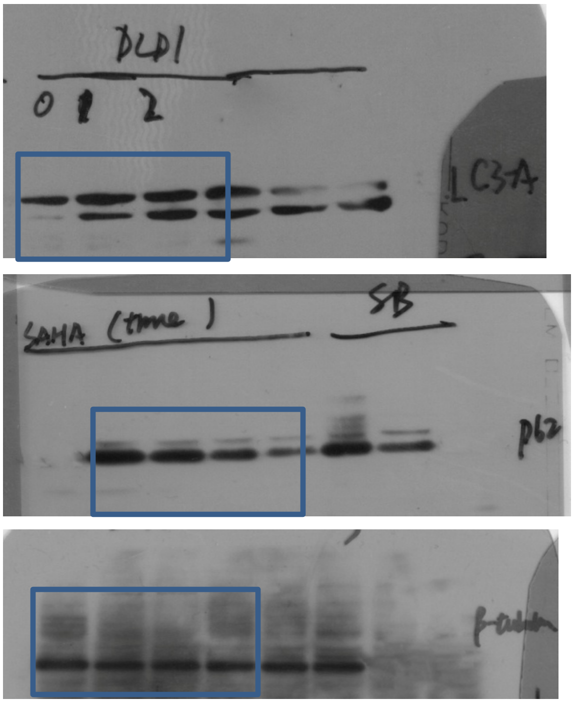


Fig.4D

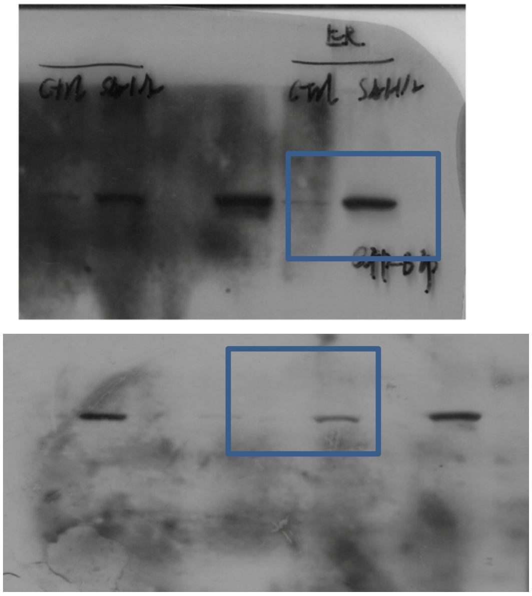

Fig.5B

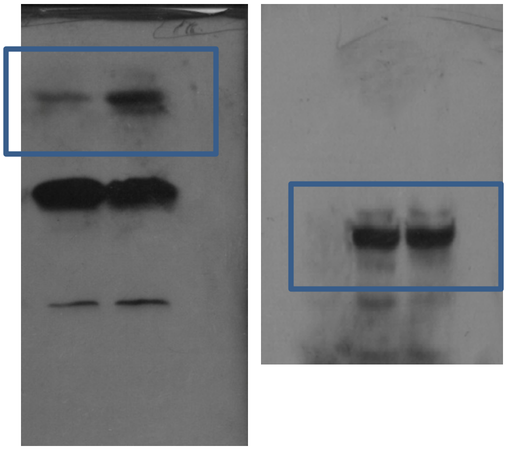

Fig.5E

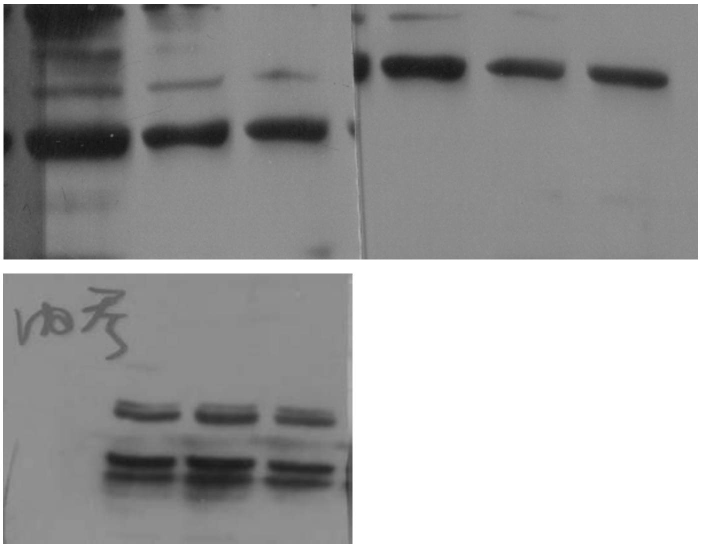

Fig. 6B

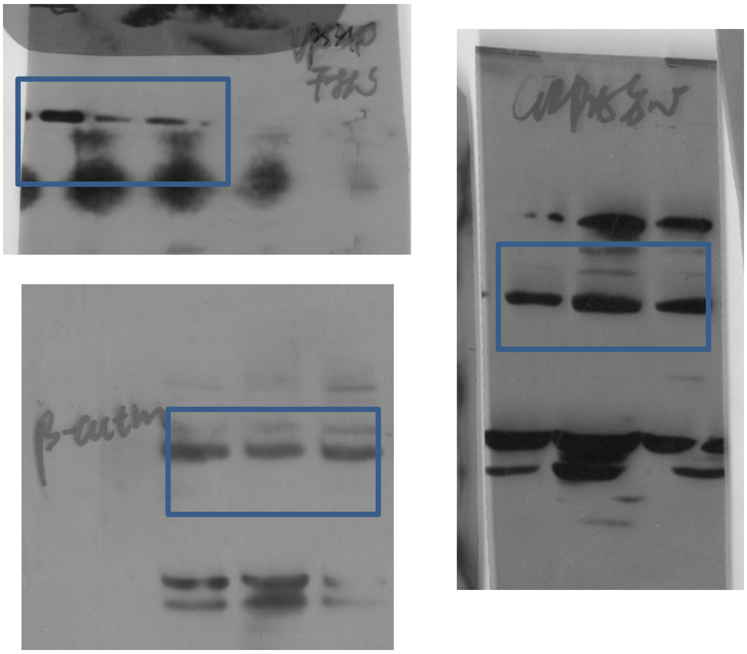

Fig.6C

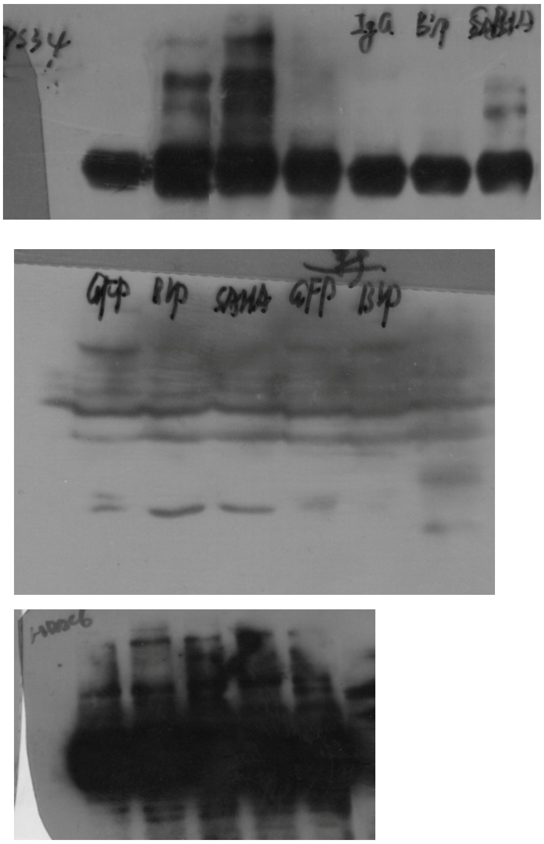

Fig. 6D

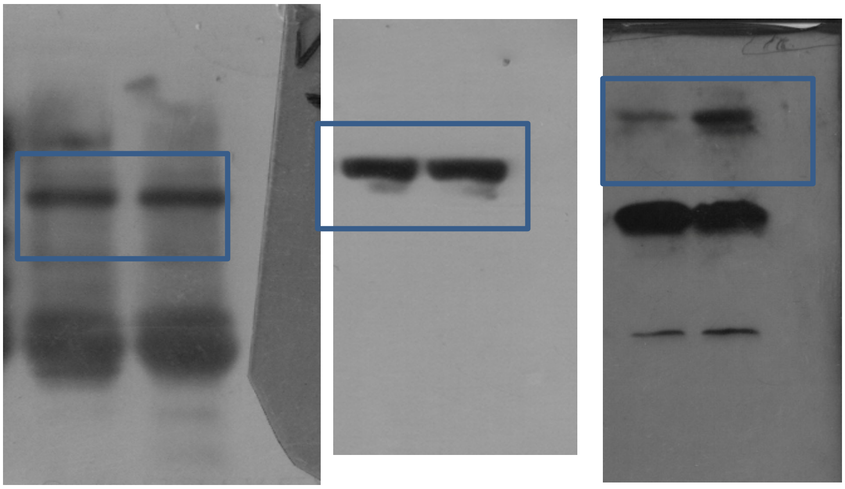

Fig.6E

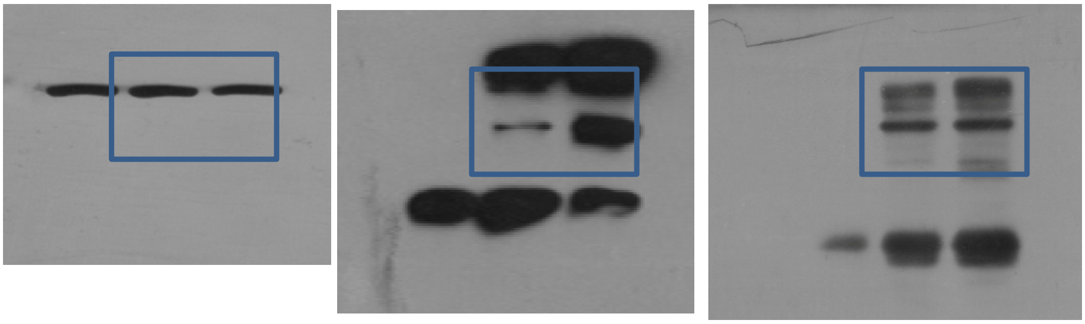

Fig.7A

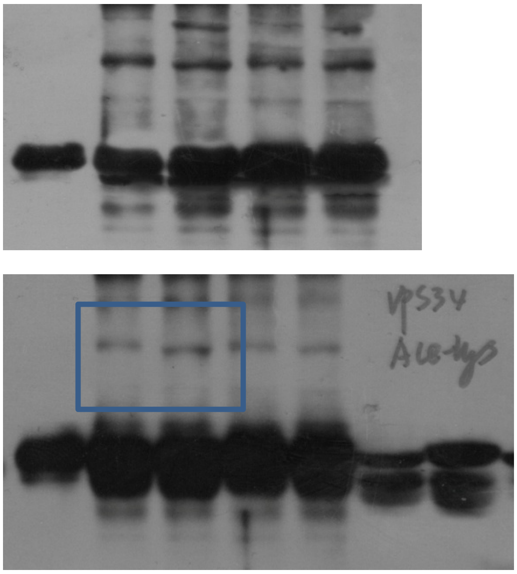

Fig.7B

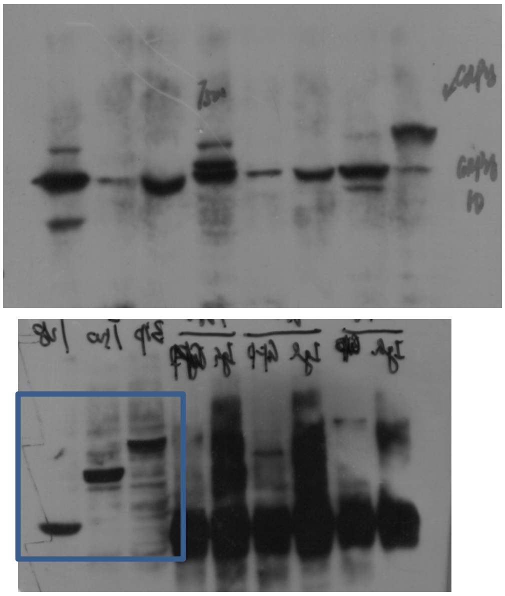

Fig.7C

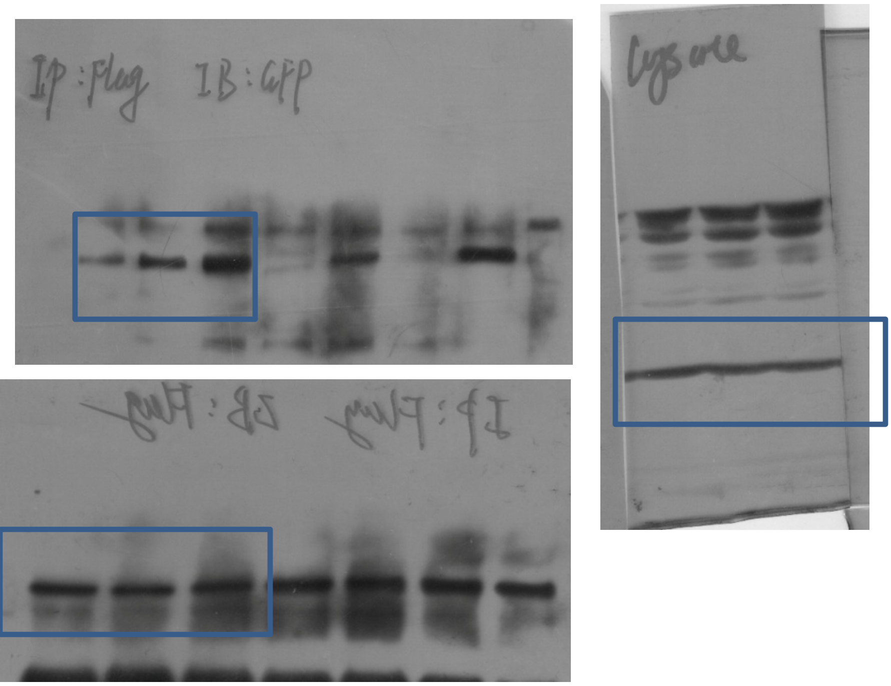


Fig. 7E

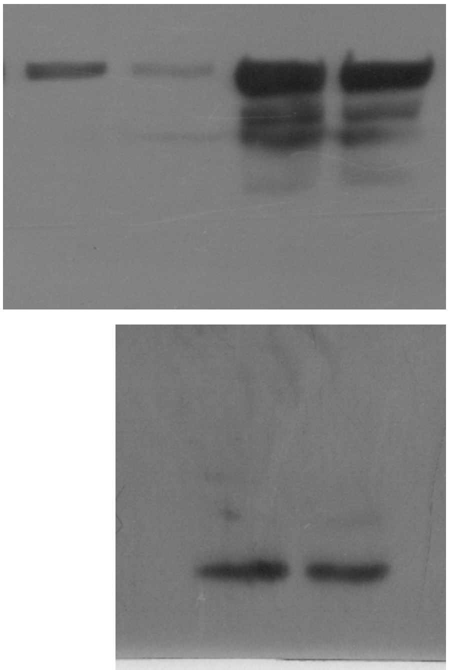

Supplement: Supplementary Information [file srep30406-s1.pdf]
